# Pharmacists’ Management of Urinary Tract Infection Symptoms in Community Pharmacy: Counseling Practices and Attitudes Toward Antibiotic Therapy

**DOI:** 10.3390/pharmacy14040100

**Published:** 2026-07-03

**Authors:** Aleksandar Jovanović, Radmila Veličković Radovanović, Ivana Tadić, Milica Drobac, Bojana Vidović, Dragana Pavlović, Marina Odalović, Dušanka Krajnović

**Affiliations:** 1 Department of Social Pharmacy and Pharmaceutical Legislation, Faculty of Pharmacy, University of Belgrade, Vojvode Stepe 450, 11000 Belgrade, Serbia; marina.odalovic@pharmacy.bg.ac.rs (M.O.); dusica.krajnovic@pharmacy.bg.ac.rs (D.K.); 2 Department of Pharmacy, Faculty of Medicine, University of Niš, Blvd. Dr Zorana Djindjica 81, 18000 Niš, Serbia; dragana.pavlovic@medfak.ni.ac.rs; 3 Department of Pharmacology with Toxicology, Faculty of Medicine, University of Niš, Blvd. Dr Zorana Djindjica 81, 18000 Niš, Serbia; radmila.velickovic.radovanovic@medfak.ni.ac.rs; 4 Department of Clinical Pharmacy, Faculty of Chemistry and Pharmacy, University of Innsbruck, Innrain 52, 6020 Innsbruck, Austria; ivana.tadic@uibk.ac.at; 5 Department of Pharmacognosy, Faculty of Pharmacy, University of Belgrade, Vojvode Stepe 450, 11000 Belgrade, Serbia; milica.drobac@pharmacy.bg.ac.rs; 6 Department of Bromatology, Faculty of Pharmacy, University of Belgrade, Vojvode Stepe 450, 11000 Belgrade, Serbia; bojana.vidovic@pharmacy.bg.ac.rs

**Keywords:** antimicrobial stewardship, community pharmacy, interprofessional collaboration, patient counseling, professional competencies, referral practices, self-care, Serbia

## Abstract

**Background/Objectives**: Pharmacists play a key role in managing urinary tract infection (UTI) symptoms by providing medications, self-care advice, and over-the-counter treatments, while referring patients to a doctor when necessary. This study aimed to examine the practices of community pharmacists in managing UTI symptoms and to gain insight into their attitudes toward antibiotic use for this condition. **Methods**: A cross-sectional study was conducted among community pharmacists in Serbia using a previously validated online questionnaire, assessed for content and face validity and pilot-tested among pharmacists. **Results**: A total of 430 community pharmacists participated in the study. Patients more often consulted pharmacists before visiting a doctor than after (median 5 vs. 3 per week; *p* < 0.001). For uncomplicated UTIs, pharmacists primarily recommended increased fluid intake (92.8%), herbal teas (94.7%), and food supplements (85.6%), whereas for complicated UTIs, most referred patients to a doctor (95.4%). Attitudes, perceived competence, and support for over-the-counter antibiotic availability were significantly associated with gender, years of experience, and specialization. Pharmacists who agreed that antibiotics are the most effective treatment for uncomplicated urinary tract infections were more likely to refer patients to a doctor (*p* < 0.01). **Conclusions**: Pharmacists are frequently consulted for UTI management and emphasize non-antibiotic approaches for uncomplicated cases. Their attitudes influence counseling practices, highlighting the need for standardized UTI counseling services, antimicrobial stewardship education, and structured communication training to support appropriate antibiotic use.

## 1. Introduction

Urinary tract infections (UTIs) are a common medical condition with varying severity and frequency. The introduction of bacteria into the urinary tract can cause inflammation and a wide range of symptoms, including painful and frequent urination, a burning sensation, fever, sepsis, and, in severe cases, death. These symptoms often cause significant physical discomfort and negatively affect patients’ quality of life. Consequently, individuals frequently seek medical advice from doctors and pharmacists [1,2].

According to the European Association of Urology (EAU) guidelines, antibiotic therapy is recommended as first-line treatment for UTIs, with empirical therapy commonly used in uncomplicated cases [3]. To ensure evidence-based care, clinical guidelines are developed and regularly updated. For example, the EAU guidelines provide recommendations based on symptom severity, risk factors, and recurrence frequency. These guidelines also recommend a short course of antimicrobial therapy for patients with recurrent UTIs [3].

Historically, high rates of non-adherence to clinical guidelines have been observed in the management of UTIs [4,5,6,7]. The overuse of fluoroquinolones and the underuse of first-line antibiotics remain persistent issues. Despite ongoing improvements, irrational antibiotic prescribing continues to contribute to the rise of antimicrobial resistance [8,9]. In light of this growing concern, antimicrobial stewardship has become essential for preserving the efficacy of available antimicrobial agents. Recent advances in rapid antimicrobial susceptibility testing (AST) have enabled the identification of antimicrobial resistance within substantially shorter timeframes than conventional culture-based methods. The broader implementation of these technologies may support antimicrobial stewardship by facilitating more timely and evidence-based antibiotic selection [10,11,12]. Recent evidence highlights that antibiotics are often unnecessary for uncomplicated UTIs, as these infections are typically self-limiting and resolve within a few days without medical intervention [13,14].

Despite clear guideline recommendations, studies have shown variability in clinical practice and adherence to evidence-based treatment of UTIs across healthcare settings [15,16]. In addition, many patients initially attempt to manage their UTI symptoms by self-medication or delaying doctor consultation, further emphasizing the importance of pharmacists in the management of UTIs [17,18,19].

Community pharmacists are well-positioned to play a crucial role in UTI management [20]. As highly accessible healthcare professionals, they are often the first point of contact for many patients [21]. In many countries, including Serbia, pharmacists provide free medical advice without an appointment in addition to dispensing medications [22,23]. Their daily practice in UTI management includes patient counseling on risk factors and prevention strategies, symptom recognition, self-care measures, appropriate use of prescription and non-prescription medicines, possible adverse drug reactions, and the use of dietary supplements and herbal products [24,25]. Given their frontline role in patient care, pharmacists are responsible for assessing the severity of UTI symptoms and referring patients to a doctor when necessary. They also contribute to the optimization of antibiotic therapy, especially in situations where therapy is not in accordance with important guidelines. This significantly contributes to the rational use of antibiotics and the prevention of antimicrobial resistance. In some countries, pharmacists are also authorized to dispense certain antibiotics for UTI treatment without a prescription. Studies suggest that pharmacist-led management of uncomplicated UTIs is both effective and safe, with high levels of patient satisfaction. This service is also considered cost-effective and has the potential to enhance the efficiency of healthcare systems [20,26,27].

Despite the important role of community pharmacists in UTI management, research in this area remains limited. The considerable variation in treatment practices, which differs substantially across countries, underscores the need for further evidence to support standardized and optimized UTI management strategies. However, little is known about how community pharmacists in the Republic of Serbia counsel patients with UTI symptoms and how their attitudes towards antibiotics affect referral practices. Therefore, this study aims to examine the practices of community pharmacists in managing UTI symptoms and to gain insight into their attitudes toward antibiotic use for this condition in the Republic of Serbia.

## 2. Materials and Methods

### 2.1. Study Design

A cross-sectional survey of community pharmacists in Serbia was conducted over four months, from November 2023 to February 2024. For the purposes of the study, the authors designed a structured Serbian questionnaire, which was a previously validated online instrument assessed for content and face validity; the validation results were recently published [28]. The questionnaire was pretested with 17 pharmacists at a community pharmacy, and their responses were excluded from the main study.

### 2.2. Structure and Content of the Survey

The questionnaire comprised 33 questions divided into three main sections: pharmacists’ practices, attitudes toward counseling patients with UTI symptoms, and respondents’ demographic and pharmacy-related characteristics. It included questions on the treatment of UTI symptoms with antibiotics and herbal products. However, for the purpose of this study, only the 17 questions related to antibiotics were analyzed. The remaining 16 questions were not antibiotic-related and were therefore excluded, as the primary objective of this manuscript was to assess pharmacists’ attitudes and practices regarding antibiotic use in UTI management.

Domain 1 consisted of five questions addressing pharmacists’ practices in managing patients with UTI symptoms. These questions explored how frequently pharmacists encounter such patients each week and whether they had previously consulted a doctor. Pharmacists were also asked about the essential advice they provide to patients with UTI symptoms, differentiating between uncomplicated and complicated cases. Additionally, they were asked to estimate how many out of five patients with UTI symptoms they typically refer to a doctor for prescription therapy.

Domain 2 included four statements assessing pharmacists’ attitudes toward antibiotic use in UTI treatment. Pharmacists indicated their level of agreement with the statement that antibiotics are the most effective and safest option for treating uncomplicated UTIs. They were also asked whether they considered themselves competent to recommend antibiotics for uncomplicated UTIs and whether specific antibiotics (e.g., fosfomycin, nitrofurantoin) should be available over the counter for this indication. Furthermore, professional attitudes toward UTI treatment were evaluated on a five-point Likert scale from 1 (strongly disagree) to 5 (strongly agree).

Domain 3 comprised eight questions on the socio-demographic characteristics of pharmacists (age, gender, specialist training, and work experience) and pharmacy characteristics (location, type, proximity to a healthcare facility, and average number of patients per pharmacist).

For the purpose of the questionnaire, the definitions of uncomplicated and complicated urinary tract infections were based on the EAU Guidelines on Urological Infections. Uncomplicated UTIs were defined as acute, sporadic or recurrent lower (uncomplicated cystitis) and/or upper (uncomplicated pyelonephritis) urinary tract infections occurring in non-pregnant women with no known relevant anatomical or functional abnormalities of the urinary tract or comorbidities. Complicated UTIs were defined as all urinary tract infections not classified as uncomplicated [3].

### 2.3. Study Population

The target population consisted of pharmacists working in community pharmacies providing primary healthcare services in the Republic of Serbia. The criteria for inclusion of respondents in the study were possession of a valid license from the Pharmaceutical Chamber of Serbia, employment in a community pharmacy, and voluntary participation in the research. Pharmacists working in hospital pharmacies, those without a valid license or those who did not provide voluntary informed consent were excluded.

### 2.4. Data Collection

At the time of the study, there were 7180 licensed pharmacists in the Republic of Serbia. All pharmacists were invited to participate in the study via an email sent by the Pharmaceutical Chamber of Serbia. The minimum sample size was 365, calculated using SurveyMonkey^®^ (San Mateo, CA, USA) at a 95% confidence level and a 5% margin of error, to ensure the sample was representative. Of the 7180 invited pharmacists, 430 completed the survey, yielding a response rate of approximately 6.0%. Data were collected using an electronic version of the questionnaire available in Google Docs (Google LLC, Mountain View, CA, USA) until the minimum number of respondents was reached. The data were collected completely anonymously, on a voluntary basis and without any request for personal data that could identify respondents. The respondents were not offered any incentives and could withdraw from the study at any time without providing a reason. The authenticity of the participants was ensured through analysis of matching responses and the time taken to complete the basic questionnaire. If duplicate responses were observed, these responses were excluded from further analysis. Also, responses with a completion time of less than 2 min were excluded from the analysis. Although there is no universally accepted limit for speeding, the 2 min threshold was defined a priori based on the questionnaire length and the minimum time required to read and meaningfully answer all questions. This approach is in line with established web survey methodology, in which extremely short completion times are considered indicators of careless responses and reduced data quality [29,30].

### 2.5. Data Analysis

A question regarding years of work experience was included, as prior research has indicated that professional experience can impact pharmacists’ adherence to evidence-based practice. Based on this, a categorization of patient counseling practices was conducted to further analyze the relationship between work experience and counseling approaches. The 10-year cut-off for professional experience was selected based on prior literature, in which this threshold is commonly used to distinguish early-career from more experienced pharmacists [31,32,33,34].

All data were entered and analyzed using SPSS version 20 (IBM Corp., Armonk, NY, USA). Comparisons between the two experience-based groups (less than 10 years vs. 10 years or more) were conducted for counselling practices, attitudes, and referral-related outcomes. All analyses were performed within a predefined analytical framework, using non-parametric or categorical tests depending on the variable type. Descriptive statistics were calculated for all variables, with frequencies and percentages used for categorical variables, and means with standard deviations (SD) or medians with interquartile ranges (IQR) for numeric variables.

Due to the non-normal distribution of the data, non-parametric tests, specifically the Mann–Whitney U test, were employed. The chi-square test was used to compare the distribution of categorical data between groups. The Wilcoxon Signed-Rank Test was used to assess the significance of the difference between the number of patients who visit a pharmacist after consulting a doctor and the number of patients with UTI symptoms who seek advice from a pharmacist first, without prior medical consultation. Additionally, the effect size for the Wilcoxon test was calculated to evaluate the practical significance of the difference, using the formula r = Z/√N, where z is the standardized value of the Wilcoxon test statistic (Z-score), N is the total number of pairs included in the analysis. According to Cohen’s criteria, an effect size of 0.1 to 0.3 is small, 0.3 to 0.5 is medium, and greater than 0.5 is large [35]. The relationship between specific attitudes and the practice of referring patients to a doctor for therapy was examined using Univariate Poisson Regression under the Generalised Linear Model function, as the referral outcome was treated as count data. Model assumptions were assessed, including checks for overdispersion. The significance level was set at *p* < 0.05 for all analyses.

### 2.6. Ethical Approval

The study was ethically approved by the Ethics Committee of the Pharmaceutical Chamber of Serbia (decision number 316/6-4-5). After data collection, all information was anonymized and kept confidential. Any details that could potentially identify respondents were excluded.

## 3. Results

The study included 430 pharmacists. The average age of participants was 40.03 years (SD = 10.40), with a median practice duration of 13.07 years. Pharmacists were categorized into two groups based on their years of experience: Group 1 (G1) included those with less than 10 years of experience, while Group 2 (G2) included those with 10 or more years of experience. Table 1 presents the socio-demographic characteristics of the pharmacists where they practice.

Pharmacists reported that they most often provide services to 50 patients per pharmacist during a working day (median), with the number of patients ranging from 20 to 300 (IQR = 40–70). The median number of those who seek advice from a pharmacist within one week after consulting a doctor is 3 (IQR = 2–6), with a weekly range of 0 to 50 patients. The median number of patients with UTI symptoms who first seek advice from a pharmacist without prior consultation with a doctor is 5 (IQR = 3–10), with a weekly range of 1 to 45 patients. This difference is statistically significant (*p* < 0.001) and has a medium practical effect (r = 0.415).

### 3.1. Counseling

**Figure 1:**
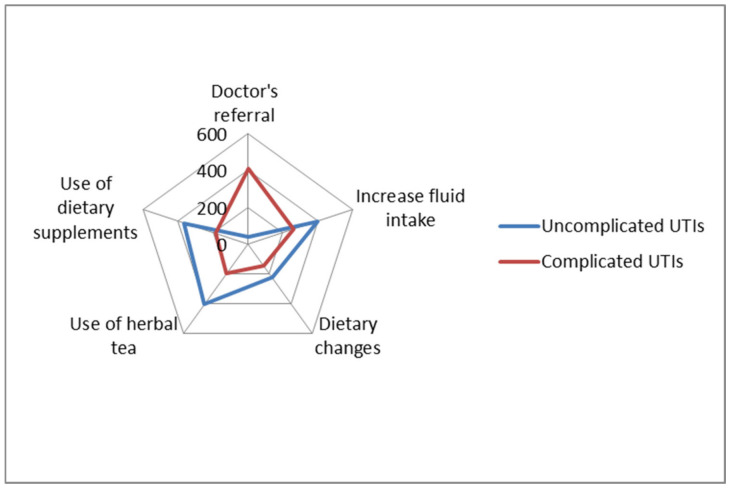
Advice routinely provided by pharmacists to patients with UTI symptoms who had not previously consulted a doctor, according to UTI severity.

Pharmacists’ counseling practices and the advice routinely provided to patients who had not previously consulted a doctor varied according to the severity of the UTI are shown (Figure 1). For patients with uncomplicated UTI, pharmacists most commonly recommend the use of herbal tea (*n* = 407, 94.65%), increased fluid intake (*n* = 399, 92.79%), use of food supplements (*n* = 368, 85.58%), change in diet (*n* = 225, 52.33%), while a smaller proportion refer to a doctor (*n* = 41, 9.54%). For complicated UTIs, the majority of pharmacists refer patients to a doctor (*n* = 410, 95.35%), while others provide advice on increased fluid intake (*n* = 260, 60.46%), use of herbal tea (*n* = 198, 46.05%), use of food supplements (*n* = 187, 43.49%), and change in diet (*n* = 149, 34.65%).

Counselling practices also vary according to pharmacists’ years of experience (Table 2). Pharmacists with less than 10 years of experience provide more advice to patients, particularly regarding dietary changes for those with complicated UTIs.

A small proportion of pharmacists (4.2%) provide only a single piece of advice for uncomplicated UTIs, most commonly recommending only herbal teas (1.9%) or food supplements (1.4%). The majority, however, advise increased fluid intake, along with herbal teas (89.8%) or food supplements (81.6%). Additionally, 42.3% of pharmacists offer all recommendations except referring patients to a doctor.

Regarding the counseling of patients with complicated UTIs, 25.1% of pharmacists refer patients to a doctor without providing any additional advice. However, most pharmacists offer supplementary recommendations alongside referral, primarily advising increased fluid intake (60.9%), herbal teas (45.6%), or food supplements (43.3%). A comprehensive approach, including all five counseling strategies, is provided by 17.4% of pharmacists.

**Figure 2:**
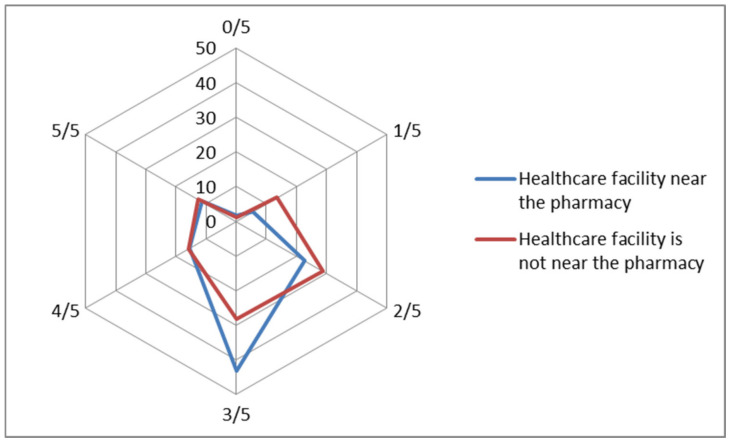
Number of patients referred to a doctor depending on the proximity of the pharmacy to the healthcare facility.

Pharmacists were asked how many out of five patients presenting with UTI symptoms they typically refer to a doctor. Only 1.4% of pharmacists reported not referring any patients, while 9.1% referred one patient, 25.3% referred two, 36.7% referred three, 15.6% referred four, and 11.9% referred all five patients. The number of patients referred to a doctor differed significantly (*p* < 0.01) by the pharmacy’s proximity to a healthcare facility (Figure 2).

### 3.2. Attitudes About Antibiotics

The majority of pharmacists either “strongly disagreed” or “disagreed” with the statement that antibiotics are the most effective treatment for uncomplicated UTIs (62.8%) and that they improve patients’ quality of life (64.0%). However, one in four pharmacists reported being neutral about their competence to recommend a specific antibiotic for uncomplicated UTIs (Table 3).

Regarding gender differences, a higher proportion of women than men reported negative views on antibiotic efficacy (63.7% vs. 54.8%, *p* < 0.05). A significantly higher proportion of pharmacists who had completed specialization in pharmacotherapy and pharmaceutical healthcare considered themselves competent to recommend a specific antibiotic compared with those without specialization (58.0% vs. 30.3%, *p* < 0.01). Additionally, pharmacists working in independent pharmacies had a more positive attitude toward recommending antibiotics than those working in larger or smaller pharmacy establishments (36.4% vs. 33.6% and 30.9%, respectively; *p* < 0.05).

Both gender and years of work experience significantly influenced attitudes toward the over-the-counter registration of certain antibiotics. Support for over-the-counter registration was reported more frequently by male than female pharmacists (42.9% vs. 28.4%, *p* < 0.05), and by pharmacists with more than 10 years of work experience compared with those with fewer years (32.7% vs. 25.6%, *p* < 0.05).

Pharmacists’ attitudes towards antibiotics and their association with the number of patients referred to a doctor for therapy were analyzed using univariate Poisson regression (Table 4). Attitudes regarding the effectiveness of antibiotics and their impact on quality of life were significantly associated with the number of reported referrals pharmacists made. Pharmacists who “strongly agree” with the statement that antibiotics are most effective in treating uncomplicated UTIs are expected to refer 30.6% more patients to a doctor compared to those in the reference group (“strongly disagree”). Similarly, pharmacists who “strongly agree” that antibiotics improve the quality of life for patients with UTIs are expected to refer 28.3% more patients to a doctor than the reference group (“strongly disagree”).

## 4. Discussion

This study revealed the significant role of Serbian pharmacists as key healthcare providers in triaging patients with UTI symptoms, offering appropriate advice and referring individuals to doctors when necessary. Similar trends were observed in a 2020 study [36], which reported that pharmacists frequently serve as primary sources of medical advice, particularly for UTIs. Although conducted in a different healthcare system, this study highlights a similar pattern of pharmacists serving as the first point of contact for patients with UTI symptoms. The reason is that patients often seek to avoid long waiting times and the costs associated with formal clinic visits, while still receiving adequate advice on treating UTI symptoms [36].

The results indicate that pharmacists commonly recommend increased fluid intake, herbal teas, and food supplements for managing uncomplicated UTIs. This aligns with evidence from prior studies, which suggest that structured hydration interventions can reduce the incidence of UTIs requiring antibiotics by up to 58% among care home residents [37]. Additionally, dietary changes, use of herbal teas and food supplements have been shown to alleviate UTI symptoms. Notably, pharmacists with less than 10 years of experience were more likely to advise on nutritional interventions, suggesting generational differences in training. Younger pharmacists have received education in dietetics, herbal medicine, and evidence-based pharmacy, including critical appraisal of scientific literature, which may explain these differences [38,39,40]. Previous research has shown that newly qualified pharmacists are more likely to rely on primary sources such as randomized controlled trials and systematic reviews, potentially influencing their clinical decision-making [41]. These findings highlight the need for ongoing education in evidence-based pharmacy, particularly for older pharmacists.

The findings indicate that pharmacists frequently encounter patients whose treatment is managed by doctors. On the other hand, pharmacists refer some patients to doctors to obtain a prescription for UTI treatment. This highlights the importance of interprofessional collaboration in optimizing patient outcomes [42,43]. Several studies emphasise the need to monitor this collaboration to ensure consistent and effective treatment. A key aspect of this process is ensuring that patients receive uniform advice from different healthcare professionals [44,45]. Our results support this notion, showing that pharmacists working in close proximity to healthcare institutions are more likely to refer patients to doctors, potentially reflecting structural differences in access to physicians and patterns of interprofessional collaboration. In addition, referral decisions are likely influenced by multiple factors, including clinical presentation, guideline recommendations, and professional judgment. Other potential contributing factors may include healthcare professionals’ confidence in their clinical decision-making (self-efficacy), which may affect their willingness to initiate treatment or referral actions. Conversely, clinical or therapeutic inertia may contribute to delays or reluctance in recommending treatment modifications or referrals when clinically indicated [46,47,48].

Pharmacists rarely refer patients with uncomplicated UTIs to doctors for antibiotic treatment, raising concerns about adherence to clinical guidelines, such as those issued by the EAU, which recommend antibiotics as first-line therapy for these infections [3,24]. Although non-antibiotic strategies have been shown to reduce overall antibiotic consumption by up to 63%, they may also lead to higher rates of incomplete recovery, subsequent antibiotic use, and an increased risk of complications, especially in patients with positive urinary erythrocytes and urine culture [49]. However, the risk of uncomplicated UTIs progressing to pyelonephritis remains relatively low (1–2%) [50]. Some studies suggest that over 30% of uncomplicated UTIs are self-limiting and that antibiotic treatment is often unnecessary, contributing to antimicrobial resistance [50,51,52]. Additionally, many patients prefer to try non-antibiotic treatments before resorting to antibiotics [53]. This finding should be interpreted with caution, as not all patients with mild or self-limiting symptoms necessarily require referral; however, referral remains important when warning signs or risk factors for complicated infection are present. Given these complexities, pharmacists must carefully balance the benefits and risks of different treatment strategies and make evidence-based decisions, highlighting the need for continuous professional education in this area.

Studies have shown that clinical outcomes for antibiotic treatment of UTI are better than with placebo. However, most pharmacists in this study expressed skepticism about the superiority of antibiotics for uncomplicated UTIs. The underlying reasons for this perspective require further investigation. One possible explanation is the frequent prescription of antibiotics that do not align with current clinical guidelines for this indication [54]. Additionally, the growing threat of antimicrobial resistance diminishes the effectiveness of antibiotics in treating UTIs [55,56], prompting increasing advocacy for therapeutic strategies that incorporate non-antibiotic measures [57]. Furthermore, antibiotic treatment has been associated with an elevated risk of recurrent UTIs, which may contribute to healthcare professionals’ reluctance to promote indiscriminate antibiotic use [50].

A significant proportion of Serbian pharmacists do not consider themselves competent to recommend a specific antibiotic for the treatment of UTIs. Similar findings have been reported in other studies, where pharmacists cited insufficient information, such as limited knowledge about the patient and a lack of clinical expertise, as barriers to providing optimal care [36]. In contrast, 75% of Arab pharmacists believe that community pharmacists are qualified to prescribe antibiotics for patients presenting with symptoms of bacterial infections [58]. In this context, emerging diagnostic innovations, such as AST, may be relevant to clinical decision-making in UTI management. For community pharmacists, such developments could support more confident, evidence-based counselling of patients with UTI symptoms by reducing uncertainty about antibiotic therapy [10,11,12]. However, the impact of these technologies on pharmacists’ attitudes and routine practice in Serbia has not yet been evaluated.

Numerous studies highlight the critical role of community pharmacies and pharmacists in outpatient antimicrobial stewardship [36,59], particularly in managing uncomplicated UTIs, which pharmacists often treat effectively and safely. Consequently, some countries have implemented policies allowing the dispensing of specific antibiotics without a doctor’s prescription for mild infections [20,60,61]. In Serbia, however, the sale of antibiotics without a prescription remains illegal. Only one-third of Serbian pharmacists believe that certain antibiotics should be available without a doctor’s prescription. Research suggests that concerns about the potential contribution of non-prescription antibiotic dispensing to antimicrobial resistance may underlie this cautious stance among Serbian pharmacists.

The findings indicate that pharmacists with specialized training in pharmacotherapy and pharmaceutical healthcare demonstrate greater confidence in recommending antibiotics, suggesting that additional education enhances professional competence. Furthermore, pharmacists with more than 10 years of experience are more supportive of reclassifying certain antibiotics as over-the-counter (OTC) medications. This may be attributed to their accumulated expertise, confidence, and practical experience in patient counseling acquired over years of practice [62].

A key finding is the significant association between pharmacists’ beliefs about antibiotic effectiveness and their likelihood of referring patients to a doctor. These results should be interpreted as associations between pharmacists’ attitudes and their self-reported referral practices, rather than as evidence that referral decisions are determined directly by personal beliefs alone. In addition, referral decisions are likely influenced by multiple factors, including clinical presentation, guideline recommendations, and professional judgment. Pharmacists who strongly believe in the efficacy of antibiotics for uncomplicated UTIs are more likely to refer patients for antibiotic therapy. These findings emphasize the need for ongoing education and training in evidence-based UTI management, ensuring pharmacists are well equipped to balance antimicrobial stewardship with appropriate patient care.

These results indicate that pharmacists in Serbia may not yet be prepared to adopt certain practice changes or implement new strategies for UTI management. Consequently, pharmaceutical practice in Serbia does not align with trends observed in developed countries such as Australia, where the majority of pharmacists (71.7%) believe that their profession is prepared for further drug reclassification and the introduction of expanded services [63]. Raising awareness and enhancing Serbian pharmacists’ knowledge are essential steps in preparing the profession to integrate modern UTI treatment strategies. These efforts aim to alleviate the burden on doctors and position pharmacists as key healthcare providers in the management of uncomplicated UTIs.

### 4.1. Implications for Practice and Policy

These findings underscore the critical role of pharmacists in UTI management and highlight the need for ongoing education to ensure adherence to evidence-based practices. Pharmacy curricula and continuing professional development programs should emphasize evidence-based approaches to UTI management, particularly in distinguishing between uncomplicated and complicated cases. Strengthening pharmacists’ expertise in this area will enhance their ability to provide appropriate patient care.

Moreover, clear guidelines on when to refer patients to a doctor could help optimize treatment outcomes and support antimicrobial stewardship efforts. The results of this study should encourage community pharmacists to expand their knowledge, improve counseling practices, and adopt appropriate treatment strategies to ensure optimal patient care.

### 4.2. Limitations and Future Research

To the best of our knowledge, this is the first study in Serbia to compare pharmacists’ practices and attitudes regarding UTI treatment between two distinct groups. However, several limitations should be acknowledged. The cross-sectional design does not allow causal inference, and reliance on self-reported data introduces potential for response bias. The relatively low response rate (6.0%) may have introduced further response bias, which should be considered when interpreting the findings. Selection bias may have occurred in the voluntary online survey because pharmacists who were more interested in UTI management or antimicrobial stewardship were more likely to respond. Additionally, recall bias may have affected participants’ responses. Furthermore, we do not have precise data on the national distribution of pharmacy types in Serbia; therefore, although the sample was heterogeneous, it may not fully reflect this distribution, a limitation to consider when interpreting the findings. Nevertheless, the sample was heterogeneous and may be considered representative of the population of pharmacists.

Future research should investigate pharmacists’ actual practices through observational studies and evaluate the impact of targeted interventions on their counseling behavior. Qualitative studies could also provide a deeper understanding of the factors shaping pharmacists’ attitudes toward antibiotic use. Moreover, as this study was limited to pharmacists practicing in Serbia, future research should include pharmacists from other countries to enable cross-cultural comparisons and broader generalizability of findings.

## 5. Conclusions

Pharmacists play a crucial role in counseling patients with UTI symptoms and adapt their counseling to the complexity of the infection. They are more likely to provide counseling to patients with uncomplicated UTIs, which is particularly important given the high prevalence of self-medication in this population. As community pharmacists’ attitudes can influence their clinical practice, targeted educational interventions should be implemented to promote the appropriate use of antibiotics.

The observed variability in pharmacists’ counseling practices and attitudes toward antibiotic use highlights inconsistencies across years of experience, specialization status, and pharmacy setting, underscoring the need for reinforcement of clinical guidelines and standardization of UTI counseling services in community practice. Addressing these gaps through structured training programs, policy initiatives, and improved collaboration between pharmacists and physicians could enhance patient care and support antimicrobial stewardship, particularly in managing complex infections. Overall, the findings indicate that both clinical behavior and attitudes toward antibiotic use vary among pharmacists and are interrelated, supporting the need for standardized, guideline-based educational interventions to improve the consistency of patient care and ensure responsible antibiotic use.

## Figures and Tables

**Table 1:** Characteristics of the community pharmacists and pharmacies (*N*  =  430).

Characteristics	Years of Work Experience		
	<10 Years (*n* = 176)	≥10 Years (*n* = 254)	*p*-Value
Sex; ***n* **(%)			
Female	148 (84.1)	240 (94.5)	0.000 ^b^ *
Male	28 (15.9)	14 (5.5)	
Age, years; mean (SD)	31.35 (5.90)	46.05 (8.40)	0.000 ^a^ *
Years of work experience in a public pharmacy; mean (SD)	4.64 (2.54)	18.91 (7.81)	N/A
Specialization in pharmacotherapy and pharmaceutical healthcare; ***n* **(%)			
Yes	13 (7.4)	37 (14.6)	0.022 ^b^ *
No	163 (92.6)	217 (85.4)	
Location; ***n* **(%)			
Urban	151 (85.8)	220 (86.6)	0.946 ^b^
Suburban	20 (11.4)	28 (11.0)	
Rural	5 (2.8)	6 (2.4)	
Type of pharmacy; ***n* **(%)			
Large multiple (≥5 pharmacies)	140 (79.5)	202 (79.5)	0.131 ^b^
Small multiple (<5 pharmacies)	27 (15.3)	28 (11.0)	
Independent	9 (5.1)	24 (9.4)	
Located near a healthcare facility; ***n* **(%)			
Yes	103 (58.5)	143 (56.3)	0.647 ^b^
No	73 (41.5)	111 (43.7)	

^a^ Mann–Whitney U test; ^b^ Chi-square test; N/A—not applicable; SD-standard deviation; * Statistically significant value.

**Table 2:** Consulting practices based on pharmacists’ years of experience.

Advice	Uncomplicated			Complicated		
	Years of Work Experience			Years of Work Experience		
	<10 Years	≥10 Years	*p*-Value	<10 Years	≥10 Years	*p*-Value
Doctor’s referral, ***n*** (%)	26 (14.8%)	21 (8.3%)	0.034 *	173 (98.3%)	238 (93.7%)	0.023 *
Increased fluid intake, ***n*** (%)	169 (96.0%)	230 (90.6%)	0.031 *	118 (67.0%)	146 (57.5%)	0.045 *
Dietary changes, ***n*** (%)	103 (58.5%)	123 (48.4%)	0.039 *	80 (45.5%)	69 (27.2%)	*p* < 0.001 *
Use of herbal tea, ***n*** (%)	168 (95.4%)	239 (94.1%)	0.538	80 (45.5%)	118 (46.5%)	0.838
Use of food supplements, ***n*** (%)	149 (84.7%)	219 (86.2%)	0.650	87 (49.4%)	100 (39.4%)	0.039 *

* Statistically significant value.

**Table 3:** Pharmacists’ attitudes towards the use of antibiotics in the treatment of UTIs.

	Strongly Disagree	Disagree	Neutral	Agree	Strongly Agree
	Percentage of Respondents				
**Antibiotics are most effective for treating uncomplicated urinary tract infections**					
Whole sample (***N*** = 430)	39.8	23.0	21.1	6.5	8.6
<10 years of work experience (***n*** = 176)	36.9	24.4	22.7	8.0	8.0
≥10 years of work experience (***n*** = 254)	41.7	22.0	21.7	5.5	9.1
Chi-square test	*p* = 0.733				
**Antibiotics improve the quality of life of patients with uncomplicated urinary tract infections**					
Whole sample (***N*** = 430)	41.9	22.1	20.5	9.1	6.5
<10 years of work experience (***n*** = 176)	39.8	21.0	21.0	11.9	6.2
≥10 years of work experience (***n*** = 254)	43.3	22.8	20.1	7.1	6.7
Chi-square test	*p* = 0.520				
**Pharmacists are competent to recommend an antibiotic to treat an uncomplicated urinary tract infection**					
Whole sample (***N*** = 430)	22.8	17.9	25.8	16.7	16.7
<10 years of work experience (***n*** = 176)	17.6	21.0	27.3	19.3	14.8
≥10 years of work experience (***n*** = 254)	26.4	15.7	24.8	15.0	18.1
Chi-square test	*p* = 0.124				
**Certain antibiotics for treating urinary tract infections (e.g., fosfomycin, nitrofurantoin) could be registered as over-the-counter medicines**					
Whole sample (***N*** = 430)	35.6	14.9	19.8	14.0	15.8
<10 years of work experience (***n*** = 176)	40.3	14.2	19.9	17.0	8.5
≥10 years of work experience (***n*** = 254)	32.3	15.4	19.7	11.8	20.9
Chi-square test	*p* = 0.007 *				

* Statistically significant value.

**Table 4:** The impact of attitudes on the practice of referring patients to a doctor.

			Univariate Poisson Regression	
Attitudes		Mean (SD)	IRR (95% CI)	*p*-Value
Belief that antibiotics are most effective for treating uncomplicated urinary tract infections	Strongly disagree	2.69 (0.088)	As reference	
	Disagree	2.84 (0.111)	1.055 (0.910, 1.224)	0.478
	Neutral	3.12 (0.109)	1.158 (1.001, 1.340)	0.049 *
	Agree	3.11 (0.264)	1.158 (0.919, 1.452)	0.218
	Strongly agree	3.51 (0.207)	1.306 (1.075, 1.587)	0.007 *
Antibiotics improve the quality of life of patients with uncomplicated urinary tract infections.	Strongly disagree	2.73 (0.084)	As reference	
	Disagree	2.85 (0.116)	1.046 (0.902, 1.213)	0.554
	Neutral	3.09 (0.123)	1.133 (0.977, 1.314)	0.098
	Agree	3.13 (0.195)	1.147 (0.941, 1.398)	0.176
	Strongly agree	3.50 (0.249)	1.283 (1.033, 1.594)	0.024 *
Pharmacists are competent to recommend an antibiotic to treat an uncomplicated urinary tract infection.	Strongly disagree	2.90 (0.129)	As reference	
	Disagree	3.06 (0.131)	1.058 (0.890, 1.257)	0.525
	Neutral	2.98 (0.097)	1.029 (0.878, 1.206)	0.724
	Agree	2.89 (0.135)	0.997 (0.834, 1.192)	0.973
	Strongly agree	2.71 (0.151)	0.935 (0.779, 1.121)	0.467
Certain antibiotics used to treat urinary tract infections (e.g., fosfomycin, nitrofurantoin) could be registered as over-the-counter medicines.	Strongly disagree	3.07 (0.098)	As reference	
	Disagree	2.94 (0.154)	0.956 (0.807, 1.132)	0.604
	Neutral	2.88 (0.114)	0.938 (0.804, 1.095)	0.419
	Agree	2.67 (0.138)	0.868 (0.726, 1.039)	0.122
	Strongly agree	2.81 (0.149)	0.914 (0.773, 1.082)	0.297

* Statistically significant value; SD—standard deviation; IRR—Incidence rate ratio; 95% CI—95% Confidence Interval.

## Data Availability

The data that support the findings of this study are available from the corresponding author upon reasonable request.

## Abbreviations

The following abbreviations are used in this manuscript:

AST	Antimicrobial Susceptibility Testing
EAU	European Association of Urology
UTI	Urinary tract infection
